# Regenerative teeth induced by in vitro mesenchymal cells in mice via repressing BMP4 and activating retinoic acid/osteopontin

**DOI:** 10.1186/s13619-025-00271-9

**Published:** 2025-12-05

**Authors:** Shubin Chen, Yifan Zhao, Hongxing Chu, Qinxing Mo, Jiashu Zhang, Xiaoming Chen, Yanmei Zhang, Xiaomei Li, Di Wu, Pengfei Liu, Bo Feng, Dajiang Qin, Yaofeng Wang, Duanqing Pei, Jinglei Cai

**Affiliations:** 1 Innovation Centre for Advanced Interdisciplinary Medicine, The Fifth Affiliated Hospital of Guangzhou Medical University, Guangzhou, 510799 China; 2 Centre for Regenerative Medicine and Health, Hong Kong Institute of Science & Innovation, Chinese Academy of Sciences, Hong Kong SAR, China; 3 Department of Periodontics and Implantology, Stomatological Hospital, Southern Medical University (Guangdong Provincial Stomatological Hospital), Guangzhou, 510515 China; 4 Department of Regeneration Medicine, School of Pharmaceutical Science, Jilin University, Changchun, 130012 China; 5 Guangdong Provincial People’s Hospital Ganzhou Hospital, Ganzhou Municipal Hospital, Ganzhou, 341099 China; 6 National & Local Joint Engineering Research Center of Biodiagnosis and Biotherapy, The Second Affiliated Hospital of Xi’an Jiaotong University, Xi’an 710016, China; 7 School of Biomedical Sciences, The Chinese University of Hong Kong, Hong Kong SAR, China; 8 Laboratory of Cell Fate Control, School of Life Sciences, Westlake University, Hangzhou, 310024 China; 9 Guangzhou Key Laboratory of Enhanced Recovery After Abdominal Surgery, The Fifth Affiliated Hospital of Guangzhou Medical University, Guangzhou, 510530 China

**Keywords:** Tooth regeneration, Odontogenic potential, In vitro maintenance, Dental mesenchymal cells

## Abstract

Maintaining the odontogenic potential of dental mesenchymal cells (DMCs) in vitro remains a critical challenge in tooth regeneration research. Current culture systems fail to sustain DMC functionality beyond short-term periods, limiting their utility for tissue engineering applications. Here, we developed an optimized N2B27-based culture medium that preserves the odontogenic capacity of mouse DMCs (mDMCs) for up to 14 days with passaging—a significant improvement over conventional methods (≤ 24 h). Single-cell RNA sequencing (scRNA-seq) revealed distinct transcriptional profiles and cellular trajectories between traditionally cultured (FBS-based) and N2B27-cultured DMCs. Mechanistically, excessive BMP4 signaling in standard media suppressed odontogenesis, whereas elevated *Spp1* (osteopontin, OPN) expression in the N2B27 system enhanced regenerative potential. We demonstrate that optimal maintenance of DMC functionality requires balanced BMP4 activity and is enhanced by high OPN levels. Notably, supplementation with recombinant OPN or all-trans retinoic acid (ATRA) partially restored tooth-forming capacity in suboptimal cultures. Our findings establish a robust in vitro platform for DMC expansion while preserving odontogenic competence, advancing both mechanistic studies of tooth development and the generation of clinically relevant cell sources for bioengineered dental tissues. This work provides key insights on the features of a regenerative tooth germ and its odontogenic microenvironment for future translational applications in tooth regeneration.

**Supplementary Information:**

The online version contains supplementary material available at 10.1186/s13619-025-00271-9.

## Background

Tooth development in mammals is a complex process that relies on the intricate interplay between dental epithelium and mesenchyme. The odontogenic potential, crucial for tooth formation, resides in dental mesenchyme from the post-bud stage until birth during embryonic organogenesis. DMCs isolated from developing molar mesenchyme have been widely used to study tooth development and regeneration (Song et al. 2006; Takahashi et al. 2010; Wang et al. 2010; Angelova Volponi et al. 2013; Oshima et al. 2014). The unique ability of odontogenic DMCs to induce non-dental epithelium into enamel-forming tissue has made them valuable for generating tooth structures and associated bone in adult renal capsules (Wang et al. 2010; Angelova Volponi et al. 2013; Ohazama et al. 2004; Otsu et al. 2014). Furthermore, ex vivo expanded DMCs from deciduous teeth have shown promise in regenerating dental pulp with an odontoblast layer, blood vessels, and nerves in both animal models and human clinical applications for tooth injuries (Xuan et al. 2018).

Our previous work has demonstrated successful whole-tooth regeneration using mouse DMCs (mDMCs) in combination epithelial sheets (Cai et al. 2009), and later with human pluripotent stem cell-derived sheets (Cai et al. 2013). Recently, we decoded subpopulations of mDMCs via scRNA-seq, revealing heterogeneities and unique profiles of odontogenic DMCs during the post-bud stage (E13.5-E16.5) (Wang et al. 2022). Despite these advances, maintaining the odontogenic potential of DMCs in vitro remains a significant challenge. Traditional culture media containing fetal bovine serum (FBS) or serum-free media with Knockout Serum Replacement (KSR) have only maintained the odontogenic potential of mDMCs for 24 and 48 h, respectively (Zheng et al. 2016). Currently, there are no adequate culture systems for the long-term maintenance of human odontogenic mesenchymal cells.

In this study, we aimed to address this critical gap by developing a novel culture medium to maintain the odontogenic potential of mDMCs in vitro. Our approach significantly extended the maintenance of odontogenic potential from 24h–48 h to at least 14 days, even after passaging, achieving a tooth induction rate of 40.74%. To elucidate the molecular mechanisms underlying this extended maintenance, we performed both bulk RNA-seq and large-scale scRNA-seq, which revealed differential gene expression profiles and distinct cell trajectories during in vitro differentiation under traditional and new culture conditions. Furthermore, we investigated the roles of the BMP signaling pathway and mineralization-related genes (e.g., *Spp1*) in preserving odontogenic potential. This study represents a significant advance in maintaining and studying odontogenic potential in vitro, opening new avenues for tooth regeneration research and potential clinical applications.

## Results

### A novel medium maintains the odontogenic potential of mDMCs for up to 14 days

**Fig. 1:**
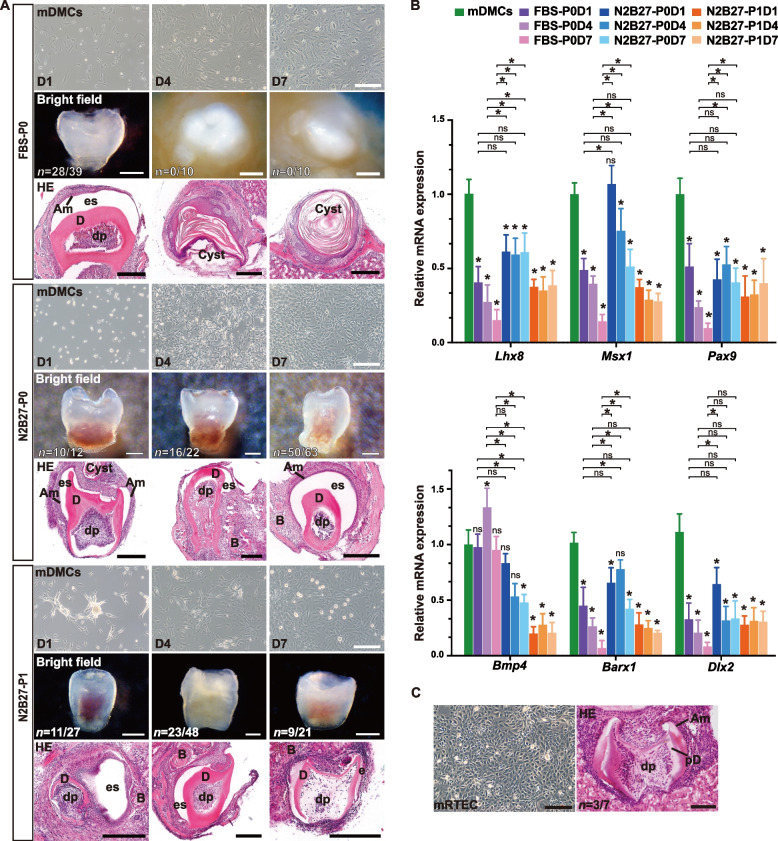
Comparison of the two culture systems for maintaining the odontogenic potential of mDMCs. **A** Morphological changes of cultured mDMCs and tooth formation efficiency at various time points in both systems. The productions were identified via bright field before sectioning and HE staining after sectioning. D, dentin; dp, dental pulp; Am, ameloblasts; es, enamel space; B, bone. Scale bar: Cells morphology = 200 μm, Bright field = 200 μm, HE = 500 μm. **B** The mRNA expression levels of dental mesenchyme-related genes in both culture systems. Freshly isolated dental mesenchyme served as a positive control. Data are expressed as the mean ± standard deviation (SD). * *P* < 0.05, ns = not significant. **C** Left: mRTECs exhibit a cobblestone-like morphology. Right: HE staining of relative tooth sections showing the tooth-like structures formed from the recombinants between mDMCs cultured for 4 days in N2B27 medium and nonodontogenic mRTECs. dental pulp (dp), predentin (pD), and ameloblast layer (Am). The scale bar correspond to 200 μm

mDMCs were isolated from E14.5 dental mesenchyme by dissociation into single cells (Fig. S1A). To extend the odontogenic potential of mDMCs, we applied a new medium supplemented with N2 and B27 components (N2B27), which are widely used for culturing neural cells (Han et al. 2020). A traditional medium containing FBS served as a control (Fig. S1A). We first assessed the duration of odontogenic potential maintenance in vitro for mDMCs cultured in both media (Fig. 1A). In FBS medium, mDMCs exhibited strong adherence and multiple pseudopods with a pronounced three-dimensional morphology upon initial attachment. By day 4 (D4), most cells displayed large, flat cell bodies. In contrast, mDMCs in N2B27 medium showed lower adherence after 24 h, with elongated cell bodies and multiple pseudopods. Following the first passage, most cells adopted a spindle-shaped, fibroblast-like morphology.

To assess the impact of culture conditions on cell viability and proliferation, a cell counting kit-8 assay was performed (Fig. S1B). Cells maintained in FBS medium following the first passage (FBS-P1) exhibited significantly reduced viability compared to the other three groups. In contrast, the other three treatment groups demonstrated consistently higher viability over the initial 5-day period. Notably, non-passaged cells in FBS medium (FBS-P0) showed a comparatively slower proliferation rate after five days, while the cultured cells in N2B27 medium still showed a stable cell proliferation efficiency. By flow cytometry, the DNA content profiles of cultured mDMCs in FBS or N2B27 medium were characteristic of a healthy, actively cycling cell population, with clear definition of the G0/G1, S, and G2/M peaks (Fig. S1C). Moreover, we performed senescence-associated beta-galactosidase (SA-β-gal) staining (Fig. S1D) and senescence-associated gene expression analysis (Fig. S1E) on mDMCs cultured in FBS or N2B27 medium at D1, D4, and D7. mDMCs treated with doxorubicin (Dox) for 48 h in FBS medium were cultured for seven days, which was used as a positive control of SA-β-gal activity, while mDMCs treated with DMSO was used a negative control (Fig. S1D). As results, cells cultured in FBS medium at D7 exhibited minimal SA-β-gal staining and no SA-β-gal activity was detected in the other groups (Fig. S1D). Molecular characterization by qPCR showed a lower and similar expression level of core senescence regulator *Cdkn2a* (*p16*) in all experimental groups with that of the negative control group (mDMCs + DMSO D7), while the core senescence regulator *Cdkn1a* (*p21*) was upregulated in the cultured cells at D4 and D7 than cultured cells at D1 in FBS medium (Fig. S1E). The upregulation of *Cdkn1a* was delayed until D7 in the cells cultured in N2B27 medium. All experimental groups showed much lower expression levels of both markers than that of the positive control group (mDMCs + Dox D7). Meanwhile, *Lmnb1* was not significantly downregulated in all experimental groups as expected, showing higher expression levels than that of positive control group (Fig. S1E). Collectively, although mDMCs cultured for seven days in vitro in either FBS or N2B27 medium showed no overt signs of senescence, the N2B27 condition significantly enhanced cell survival compared to passaged FBS cultures.

To evaluate their odontogenic potential, we performed classical tissue recombination assays using non-odontogenic dental epithelium (E14.5-E16.5). In the FBS group, tooth-like structures were generated only from mDMCs cultured for 24 h (P0D1) (71.79%, *n* = 28/39, Fig. 1A). mDMCs cultured for four or seven days formed only cysts upon recombination. In contrast, the N2B27 groups exhibited tooth-like structure formation from cultured mDMCs at both P0 (without passage) and P1 (after the first passage). The tooth formation rates at P0 were 83.33% (D1, *n* = 10/12), 72.73% (D4, *n* = 16/22), and 79.37% (D7, *n* = 50/63), and at P1, they were 40.74% (D1, *n* = 11/27), 47.92% (D4, *n* = 23/48), and 42.86% (D7, *n* = 9/21). However, mDMCs did not proliferate sufficiently after the second passage (P2). Most of the obtained tooth-like structures contained typical dental features, including an enamel-secreting ameloblast layer, enamel (space) or pre-enamel secretion, dentin, and dental pulp, often surrounded by bone (B) and possible associated cysts (Fig. 1A). Collectively, mDMCs cultured in FBS medium lose their ability to generate tooth-like structures beyond 24 h, whereas the N2B27 medium effectively maintains the odontogenic potential of mDMCs for up to 14 days, including through one passage.

We next investigated the expression of mDMCs markers (*Lhx8*, *Msx1*, *Pax9*, *Bmp4*, *Barx1*, *Dlx2*) using freshly isolated E14.5 mDMCs as a positive control (Fig. 1B). In the FBS group, the expression levels of five odontogenic genes (excluding *Bmp4*) were significantly lower at all time points (D1, D4, and D7) compared to those in the control group. These five genes showed a progressive decrease in expression with prolonged culture time in the FBS group. Conversely, the N2B27 groups exhibited markedly higher expression levels of these five genes at all time points of both P0 and P1 compared to the FBS groups at D4 and D7. The effects of the two culture media across different time points at the P0 stage (D1, D4, and D7) were analyzed using a two-way analysis of variance (ANOVA), followed by a post-hoc test for specific pairwise comparisons. The results showed that the expression levels of *Lhx8*, *Msx1*, *Pax9*, and *Barx1* was significantly downregulated over time in FBS culture compared to those in N2B27 culture (excluding *Bmp4* and *Dlx2*). Although the expression levels of five odontogenic genes (excluding *Bmp4*) was significantly downregulated in cultured mDMCs compared to fresh mDMCs, the N2B27 group demonstrated a greater ability to maintain the expression of these genes relative to the FBS group. Interestingly, *Bmp4* maintained similar or even higher expression levels in the FBS groups compared to both the control and N2B27 groups. Furthermore, the expression level of the *Dlx2* gene was significantly down-regulated after in vitro culture in both the FBS and N2B27 groups, with no significant difference between the two culture systems. This suggests that *Dlx2* may play a role in the early stages of tooth germ development. Notably, the expression levels of *Lhx8*, *Msx1*, *Barx1*, and *Pax9* in the N2B27 groups at both P0 and P1 were higher than those in FBS groups that had lost odontogenic potential (P0D4 and P0D7). These data suggest that moderate Bmp4 expression may contribute to maintenance of the odontogenic potential of mDMCs in vitro.

To confirm whether mDMCs cultured in N2B27 medium could induce odontogenesis with non-dental epithelial components, we used the mouse renal tubular epithelial cell line. The mouse renal tubular epithelial cells (mRTECs) were positive for the mRTEC-specific marker *Nphs1* and the general epithelial markers *Krt8* and *Pitx2*, but negative for the odontogenic-specific epithelial marker *Fgf8* and mesenchymal marker *Msx1* (Fig. S1F). Recombinants consisting of N2B27-treated cell pellets on D4 (P0D4) and mRTEC sheets produced the tooth-like structures with hard tissues when transplanted into adult renal capsules after three weeks, with an odontogenic ratio of 42.9% (*n* = 3/7) (Fig. 1C).

In summary, mDMCs cultured in N2B27 medium demonstrate higher odontogenic potential than those cultured in FBS medium, express elevated levels of odontogenic markers, and exhibit enhanced ability to induce odontogenesis with non-dental epithelial components.

### Transcriptome profiling reveals distinct gene expression patterns

**Fig. 2:**
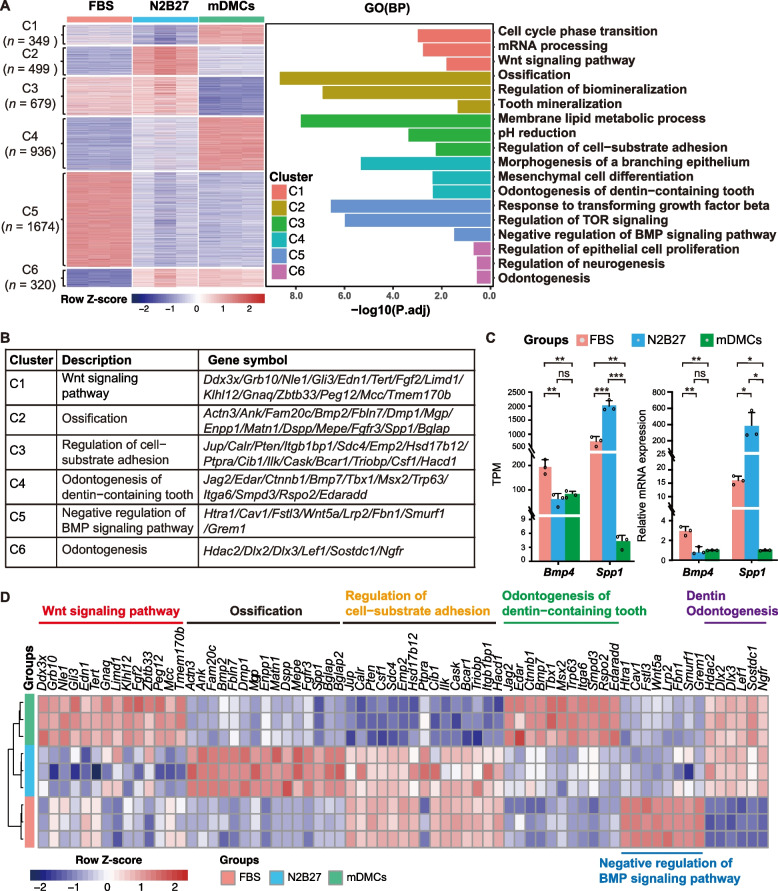
Transcriptome analysis of mDMCs samples from the two culture systems. **A** Left: Heatmap of six differentially expressed gene (DEG) clusters between mDMCs cultured in FBS medium and N2B27 medium; mDMCs, freshly isolated dental mesenchyme used as a positive control. Right: GO analysis of the six DEG clusters. **B** Representative genes of important GO terms in the six DEG clusters. **C** Bar plot showing gene expression levels of *Bmp4* and *Spp1*. Data are expressed as the mean ± SD. * *P* < 0.05, ** *P* < 0.01, *** *P* < 0.001, ns = not significant. **D** Heatmap of genes associated with key GO terms and signaling pathwas

Bulk RNA-seq results revealed distinct transcriptome profiles between mDMCs cultured in FBS and N2B27 media (Fig. 2A). We clustered the activated/silenced genes among the groups. Two important gene clusters (C2, *n* = 499; C6, *n* = 320) were identified to be activated in N2B27-cultured mDMCs. Gene ontology (GO) analysis of C6 showed enrichment of genes related to epithelial proliferation regulation, neurogenesis, and odontogenesis (*Hdac2*, *Dlx2/3*, *Ngfr*, *Sostdc1*; Fig. 2B-D), which were activated in N2B27-cultured cells and freshly isolated E14.5 mDMCs (control) but silenced in FBS-cultured mDMCs. C2 genes were highly associated with ossification and biomineralization (*Fam20c*, *Dmp1*, *Dspp*, *Spp1*; Fig. 2B, D) and were specifically expressed in N2B27-cultured mDMCs. A cluster of genes (C5, *n* = 1,647; Fig. 2A) specifically activated in FBS-cultured mDMCs was enriched for negative regulators of BMP signaling (*Htra1*, *Cav1*, *Wnt5a*; Fig. 2B-D). These findings suggest that odontogenic and biomineralization genes are activated in N2B27-cultured mDMCs.

**Fig. 3:**
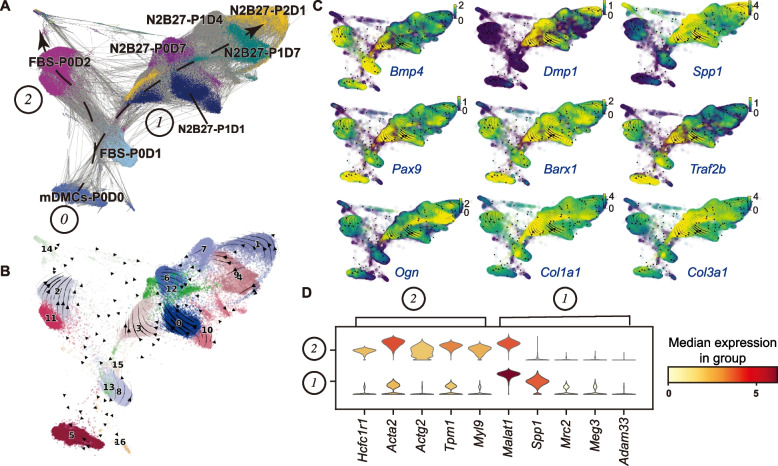
Single-cell transcriptome analysis of mDMCs samples from the two culture systems. **A** Cell trajectories of mDMCs cultured in FBS medium and N2B27 medium respectively, illustrated on a Force Atlas plot. Cell sub-populations are colored by samples. **B** RNA velocity inference of mDMCs cultured in FBS medium or N2B27 medium to predict their future transcriptional states, illustrated on a Force Atlas plot. The velocity of gene expression can be represented as mRNA abundance over time, which enables the prediction of the future transcriptional state of cells (arrows denote directionality). Cell sub-populations are colored by Leiden cell clusters. **C** Gene expression levels of representative genes. **D** Violin plot showing the expression levels of critical genes between cell path1 and path2

Next, to investigate the high-resolution trajectory of cultured mDMCs in both media, we performed scRNA-seq of eight samples (Fig. S1A, Fig. 3A): freshly isolated mDMCs (mDMCs-P0D0), FBS-treated cells (FBS-P0D1 and FBS-P0D2), and N2B27-treated cells (N2B27-P0D7, N2B27-P1D1, N2B27-P1D4, N2B27-P1D7, and N2B27-P2D1). A total of 69,867 cells passed quality control and were analyzed. The cell trajectory analysis revealed two distinct paths (Fig. 3A): an odontogenic path (path1) including FBS-P0D1 and N2B27-treated cells in a time-sequential manner, and a non-odontogenic path (path2) composed of FBS-P0D2 cells. Unsupervised clustering analysis identified 16 cell clusters (Fig. 3B). *Pax9* and *Barx1* expression levels were maintained in both cell paths. However, *Dmp1*, *Spp1*, *Traf2b*, *Col1a1*, and *Col3a1* were specifically activated in the early stage of path1, whereas *Ogn* was activated in the late stage (Fig. 3C). Marker gene analysis revealed that *Malat1*, *Spp1*, *Mrc2*, and *Meg3* were the most specific genes in cell path1, whereas *Hcfc1r1*, *Acta2*, *Actg2*, *Tpm1*, and *Myl9* were specific to cell path2 (Fig. 3D). Notably, *Bmp4* was highly expressed in cell path2 but not in path1, suggesting that BMP signaling inhibits the odontogenic potential under FBS medium culture conditions (Fig. 3C).

### BMP signaling regulates the odontogenic potential of cultured mDMCs

**Fig. 4:**
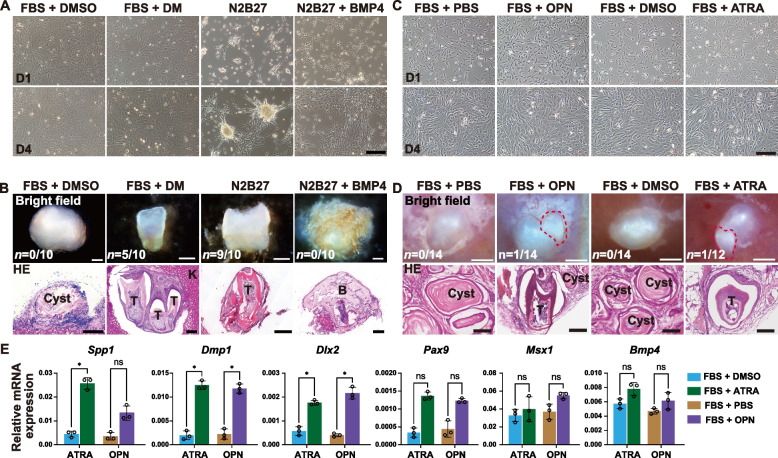
Roles of BMP4, OPN and ATRA in maintaining the odontogenic potential. **A** Cell morphology and changes over four days in the two culture media and the addition of BMP4 or a BMP4 inhibitor. Scale bar: 200 μm. **B** Tooth formation efficiency via bright field imaging and HE staining. T, tooth; K, kidney; B, bone. Scale bar: Bright field and HE = 200 μm. **C** Cell morphology and changes over four days in the FBS medium with the addition of OPN or ATRA. Scale bar: 200 μm. **D** Tooth formation efficiency via bright field imaging and HE staining. T, tooth; Scale bar: Bright field = 500 μm, HE = 200 μm. **E** Relative mRNA expression levels of key odontogenic and mineralization genes on mDMCs after treatment OPN or ATRA in FBS culture system for four days. FBS medium supplemented with PBS (FBS + PBS) served as a control condition of OPN treated group (FBS + OPN). FBS medium supplemented with DMSO (FBS + DMSO) served as a control condition of ATRA treated group (FBS + ATRA). Gene expression was quantified by qPCR and normalized to *β-actin* using the 2^(-ΔΔCt) method. Data are expressed as the mean ± SD. * *P* < 0.05, ns = not significant

The negative role of BMP signaling in odontogenic maintenance was further confirmed through recombination experiments. Cultured cells at P0D4 from each group were recombined with non-odontogenic dental epithelium (E14.5–16.5) and incubated under the renal capsule for three weeks. Inhibition of BMP type I receptors with dorsomorphin (DM, 2 μM) partially rescued the odontogenic potential of cultured cells in the FBS group (*n* = 5/10). Conversely, over-activation of BMP signaling by adding BMP4 (25 ng/mL) completely blocked the odontogenic potential in the N2B27 group (*n* = 0/10) (Fig. 4A-B). These results indicate that sustained high expression of Bmp4 suppresses the odontogenic potential of mDMCs in vitro.

### Osteopontin protein and all-trans retinoic acid signals promote the odontogenic maintenance of cultured mDMCs

Our findings suggest that high expression of mineralization-related genes may contribute to the maintenance of odontogenic fate in N2B27-treated mDMCs (Fig. 3C-D). We further investigated the potential role of the mineralization-related gene *Spp1* in odontogenesis. Using recombination and transplantation techniques, we observed a tooth-like structure in 1 out of 14 recombinants (1/14) derived from cultured cells of the FBS group (P0D4) supplemented with osteopontin (OPN protein product of the *Spp1* gene, 1 μg/mL). This result indicates that *Spp1* plays a role in maintaining the odontogenic potential (Fig. 4D).

We noted that N2B27 medium contains all-trans retinoic acid (ATRA), a molecule known to play roles in morphogenesis and biomineralization (Lourenco et al. 2020). Therefore, we examined the effect of ATRA on odontogenesis. We observed a tooth-like structure produced from recombinants containing cultured cells from the FBS group (P0D4) supplemented with ATRA (Fig. 4C). Specifically, a tooth-like structure was detected in 1 out of 12 recombinants (1/12) when ATRA was added to the FBS-cultured cells (P0D4).

**Fig. 5:**
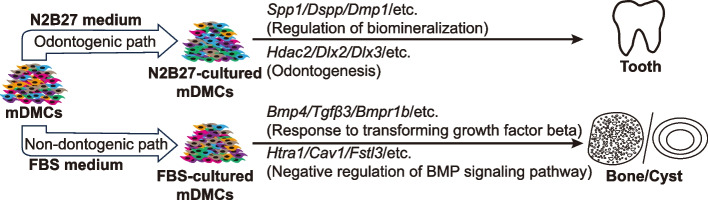
Schematic diagram of the proposed mechanism for maintaining the odontogenic potential in DMCs using the novel N2B27-based culture medium

To assess the osteo/odontogenic potential of OPN- or ATRA-treated mDMCs at the genetic level, we conducted qPCR analysis of key regulators or markers involved in tooth development and mineralization (Fig. 4E). Relative to untreated controls, cells treated with OPN showed significant upregulation of *Dmp1* and *Dlx2*. Similarly, ATRA treatment led to marked increases in *Dmp1*, *Dlx2* and *Spp1* expression. Among the genes analyzed, *Bmp4* and *Msx1* were expressed at relatively high levels in control cells; however, no significant differences were detected between control and treatment groups. Although *Pax9* expression did not reach statistical significance, an upward trend was observed following treatment. These molecular profiles confirm the activation of tooth development-related and mineralization-related pathways, despite the variable structural outcomes observed in vivo. Collectively, these results suggest that both OPN and ATRA can enhance the odontogenic capacity of cultured mDMCs. The addition of these factors to the culture medium improves the maintenance of odontogenic potential in vitro. Integrating findings from bulk RNA-seq and scRNA-seq analyses, we observed that mDMCs cultured in N2B27 medium maintained the expression of odontogenesis-related genes while upregulating genes associated with dental mineralization, thereby preserving effective odontogenic potential. In contrast, mDMCs cultured in FBS-supplemented medium predominantly upregulated genes related to the response to transforming growth factor beta and negative regulation of BMP signaling pathways. Upon epithelial recombination and transplantation, these FBS-cultured cells primarily formed bone or cystic structures (Fig. 5). These results collectively demonstrate the differential gene expression profiles and functional outcomes between N2B27 and FBS culture conditions, highlighting the superior ability of N2B27 medium to maintain odontogenic capacity.

## Discussion

Maintaining the odontogenic potential of DMCs in vitro has been a significant challenge in the field of tooth regeneration. While some studies have demonstrated the feasibility of autologous implantation of pulp tissue or stem cells (Huang et al. 2019; Ling et al. 2020; Li et al. 2020), it is still challenging to maintain the odontogenic potential of DMCs in vitro and subsequently regenerate a whole tooth. In this study, we developed a novel culture condition using N2B27 medium that maintains the odontogenic potential of mDMCs for at least 14 days with one passage. Our results demonstrate that these cultured mDMCs can form whole teeth when combined with non-odontogenic epithelial components in mice, providing an efficient platform for investigating tooth regeneration.

The success of N2B27 medium in maintaining odontogenic potential contrasts sharply with conventional FBS-containing media, which typically lead to rapid loss of this potential (Zheng et al. 2016). Our findings indicate that the N2B27 medium fosters a more favorable microenvironment for mDMCs by modulating key signaling pathways, such as Wnt, BMP, TGF-β, and Shh based on the sequencing data. All of these pathways were established critical regulators of DMC differentiation and mineralization (Chen et al. 2009; Jussila and Thesleff 2012; Yuan et al. 2015; Liu et al. 2022; Zhang et al. 2025a, b). The ability of N2B27-cultured mDMCs to induce tooth formation when combined with non-odontogenic epithelial components indicates that these cells retain the capacity to initiate and guide epithelial-mesenchymal interactions crucial for tooth development.

In our previous study (Wang et al. 2022), using scRNA-seq of in vivo mouse dental cells from E10.5 to E16.5, we identified a small population of dental epithelial cells secreting proteins that act as important ligands or antagonists of WNT, tyrosine kinase, and the BMP signaling pathways. Corresponding receptors were highly expressed in DMCs, suggesting that these secreted proteins regulate cell fate and odontogenic potential via epithelial-mesenchymal interactions. In the present study, our results highlight the critical role of BMP signaling in regulating the odontogenic potential of mDMCs. The high expression level of *Bmp4* in FBS-cultured groups, contrasting with relatively low expression level in N2B27-cultured groups, suggests that persistent overexpression of BMP4 signaling may inhibit the odontogenic potential. This is further supported by the inhibition of tooth formation upon the addition of exogenous BMP4 to N2B27-cultured cells. These findings are consistent with previous report (Zhao et al. 2000) and extend our understanding of the role of BMP4 in odontogenesis, indicating a dose-dependent regulatory mechanism.

*Bmp4* is a critical signaling molecule initially expressed in the dental epithelium. It induces the expression of *Msx1* in the mesenchyme (Vainio et al. 1993; Tucker et al. 1998). The expression of mesenchymal-derived *Bmp4* depends on *Msx1*, while simultaneously sustaining *Msx1* expression through a positive feedback signaling mechanism (Chen et al. 1996). Our findings reveal a paradoxical pattern in FBS-cultured mDMCs: sustained high *Bmp4* expression persists despite *Msx1* downregulation. This apparent decoupling of the *Msx1*-*Bmp4* regulatory axis likely reflects compensatory *Bmp4* overexpression in dental epithelium, potentially driven by alternative pathways (e.g., FGF or Wnt signaling) that maintain *Bmp4* levels when mesenchymal feedback is disrupted, as observed in *Msx1*^−/−^ models (Chen et al. 1996; Bei and Maas 1998). The continuous high *Bmp4* exposure under these conditions may disrupt by suppressing key epithelial signals like *Shh* and *Bmp2* (Zhao et al. 2000), ultimately impairing the precise spatiotemporal coordination of BMP signaling required for successful tooth formation (Liu et al. 2022; Tucker et al. 1998).

RNA-seq data revealed distinct transcriptome profiles between mDMCs cultured in FBS and N2B27 media. The identification of gene clusters C2 and C6, which activated in N2B27-cultured mDMCs, provides insight into the molecular mechanisms underlying the maintenance of the odontogenic potential. The enrichment of genes in cluster C6 related to epithelial proliferation regulation, neurogenesis, and odontogenesis, along with ossification and biomineralization genes in cluster C2, suggests that N2B27 medium promotes a more developmentally appropriate gene expression profile. Conversely, the activation of genes that negatively regulate BMP signaling in FBS-cultured mDMCs (cluster C5) may contribute to the loss of the odontogenic potential under these conditions. Furthermore, our scRNA-seq analysis revealed two distinct cell trajectories during in vitro culture. This divergence provides a high-resolution view of the molecular changes that occur as mDMCs either maintain or lose their odontogenic potential. The identification of specific marker genes for each path, such as *Dmp1*, *Spp1*, *Traf2b*, *Col1a1*, and *Col3a1* for the odontogenic path, offers potential targets for future interventions aimed at maintaining or enhancing odontogenic potential in *vitro*.

Our findings suggest that OPN and ATRA may play roles in maintaining the odontogenic potential. In this study, bulk RNA-seq and scRNA-seq analyses revealed a distinct cluster of mineralization-associated genes, including *Spp1*, specifically activated in N2B27-cultured cells along the odontogenic trajectory. Functional validation confirmed that supplementation with recombinant OPN protein could partially restore the tooth-forming capacity of mDMCs cultured in FBS medium. This finding is consistent with the known roles of OPN in biomineralization and dental tissue repair (Saito et al. 2016; Foster et al. 2018). OPN is a highly phosphorylated sialoprotein that is a prominent component of the mineralized extracellular matrices of bones and teeth (Sodek et al. 2000), involved in regulating hydroxyapatite crystal growth and promoting cell adhesion and survival in different typies of cells (Qin et al. 2004; Singh et al. 2018; Vay et al. 2021). Our data extend this understanding by implicating OPN in the maintenance of odontogenic progenitors DMCs, possibly through enhancing cell–matrix interactions and providing pro-survival signals that preserve regenerative competence. Similarly, the addition of ATRA to FBS medium also partially rescued odontogenesis. This effect is particularly interesting given that ATRA is a component of the N2B27 formulation. Retinoic acid (RA) signaling is a well-known morphogen in embryogenesis, with complex and stage-specific effects on tooth development. Either an excess or a deficiency of RA can lead to severe dental anomalies (Punyasingh et al. 1984; Morkmued et al. 2016). However, at specific concentrations, RA has been shown to promote the differentiation of dental pulp cells and odontoblasts in vitro (Wang et al. 2020; Escobar et al. 2021). Our qPCR analysis showed that ATRA treatment upregulated key odontogenic (*Dlx2*) and mineralization genes (*Dmp1*, *Spp1*), supporting its role in activating genetic programs conducive to tooth formation. Furthermore, the interaction metabolism between ATRA and vitamin D might synergistically enhance the bioavailability of factors crucial for calcium handling and biomineralization, creating a more favorable microenvironment for odontogenesis (Schrader et al. 1993; Paukovcekova et al. 2020). The synergistic or additive potential of OPN and ATRA is an exciting avenue for future research. While each factor alone showed a modest rescue effect in our suboptimal culture system, their combined action within the N2B27 medium likely contributes to its superior performance. The N2B27 formulation, originally designed for neural stem cells, may serendipitously provide a cocktail of factors that repress inhibitory signals (like excessive BMP4) and activate promotive pathways (like OPN and RA signaling), effectively preserving a “regenerative niche” in vitro.

Recent reports provide rich resources on cellular dynamics during mouse tooth development (Hu et al. 2022; Jing et al. 2022). Mouse molar development is considered more relevant to human tooth development than other models. However, the number of embryonic odontogenic cells is quite limited and exhibits considerable diversity. Moreover, these odontogenic stem cell populations rapidly lose their “stemness” in vitro, which severely restricts their utility in tissue regeneration (Min et al. 2011). Through scRNA-seq analysis, we observed heterogeneity within mDMCs. Specifically, a subset of mDMCs cultured in N2B27 medium maintained a cell fate consistent with the odontogenic differentiation pathway. This subpopulation will be a key focus of our future research. In contrast, the majority of mDMCs cultured in FBS medium displayed altered cell fates. Our findings have significant implications for the fields of tooth regeneration and stem cell biology. The ability to maintain the odontogenic potential in vitro using N2B27 medium provides a valuable tool for studying tooth development and testing regenerative strategies. In our another study, using N2B27 medium, we identified a rare *Alx3*^+^*/Barx1*^+^ subpopulation within mouse cranial neural crest cells, which differentiated into two odontogenic clusters (*Pax9*^+^*/Bmp3*^+^ and *Lhx6*^+^*/Dmp1*^+^), enabling the induction of whole tooth-like structures (Zhao et al. 2024). Moreover, the insights from our transcriptomic and single-cell analyses enhance our understanding of the molecular mechanisms underlying odontogenic fate determination and maintenance. Future studies should aim to validate this culture system in human cells, which is critical for translating our findings into clinical applications. Additionally, the dose-dependent effects of OPN and ATRA on odontogenesis require systematic investigation to optimize their concentrations for maximal efficacy. Exploring the synergistic effects of OPN/ATRA with other signaling pathways (e.g., Wnt or FGF) could further enhance the maintenance of the odontogenic potential. Such combinatorial approaches may provide a more robust framework for scaling up cell sources in bioengineered tooth regeneration.

While this study utilized mouse embryonic DMCs, translating findings to human applications necessitates addressing the distinct characteristics and challenges of human DMCs. Human DMCs are categorized by developmental stage: embryonic (e.g., cap stage, ~ 12 weeks gestation in humans, analogous to E14.5 DMCs in mice), primary tooth-derived, and permanent tooth-derived (Lin et al. 2007; Shi et al. 2020). Research on human embryonic DMCs is severely limited by ethical and sourcing constraints. Although studies (e.g., via microarrays, laser capture microdissection, scRNA-seq) reveal gene networks and interspecies differences (Yu et al. 2024; Zhang et al. 2025a, b), a critical barrier remains: the inability to expand these cells in vitro while preserving their comprehensive odontogenic potential—the capacity to instruct non-dental epithelium towards whole-tooth formation. In contrast, postnatal DMCs (DPSCs, dental pulp stem cells; SHED, stem cells from human exfoliated deciduous teeth; DFSCs, dental follicle stem cells; PDLSCs, periodontal ligament stem cells) are more accessible and exhibit multipotency, particularly towards odontoblast/cementoblast lineages, making them attractive for regenerative strategies targeting specific tissues (dentin-pulp or periodontium) (Xuan et al. 2018; Liu et al. 2024; Zhang et al. 2020; Yang et al. 2019; Zhang and Yelick 2021). In contrast, they exhibit a more limited range of odontogenic potentials compared to the embryonic DMC. Their clinical translation faces major hurdles: (1) Dedifferentiation and senescence during necessary in vitro expansion, leading to loss of odontogenic capacity linked to epigenetic silencing of key genes (e.g., *DSPP*, *DMP1*, *BSP*) and niche disruption (Gronthos et al. 2000; Yu et al. 2010); (2) Non-physiological two-dimensional (2D) culture environments failing to replicate the complex three-dimensional (3D) in vivo niche involving extracellular matrix, signaling gradients, and epithelial crosstalk (Huang et al. 2009); (3) Significant heterogeneity and donor variability affecting potency. Future research must therefore focus on: developing biomimetic 3D culture systems (e.g., bioprinting, hydrogels, decellularized matrices) (Zhang et al. 2017; Khayat et al. 2017); creating defined media and employing small molecules (e.g., Wnt agonists, epigenetic modulators) to maintain stemness and potential (Rahman et al. 2022; Bayarsaihan 2016; Li et al. 2024); and crucially, recreating essential epithelial-mesenchymal interactions, potentially using induced pluripotent stem cells (iPSC) derived cells with embryonic-like potential within 3D scaffolds, to achieve functional bio-tooth regeneration (Otsu et al. 2011; Arakaki et al. 2012; Cai et al. 2013).

This study is limited by the observation that, even in N2B27 medium, mDMCs undergo progressive differentiation with passaging and exhibit a concomitant decline in odontogenic competence. This likely reflects the inability of conventional 2D systems to recapitulate the signaling homeostasis and epithelial-mesenchymal crosstalk that stabilize embryonic dental mesenchyme. To address this bottleneck, we envision a non-differentiation-promoting maintenance strategy along three axes. First, chemically defined, serum-free media should be employed to finely tune core pathways (FGF/WNT/BMP/TGF-β) and preserve progenitor programs (for example, combining FGF2/FGF10 and PDGF with mild WNT activation and selective TGF-β/BMP restraint), with transient rho-associated kinase (ROCK) inhibition after passaging to mitigate stress-induced differentiation and quantitative monitoring of markers such as PAX9 and MSX1 to guide optimization. Second, biomimetic 3D microenvironments with adjustable mechanics and physiological hypoxia (approximately 2%–5% O_2_)—using collagen/hyaluronic acid hydrogels or dentin/extracellular matrix derived matrices—should better maintain native metabolic and epigenetic states. Third, restoration of epithelial cues via co-culture with dental epithelium or epithelial organoids, use of epithelial conditioned media or extracellular vesicles, and controlled delivery of FGF8, SHH, and WNT signals may help sustain odontogenic potential. Complementary reporter-guided small-molecule screens could systematically map the maintenance–differentiation landscape and yield robust, batch-consistent formulations. In parallel, iPSC-derived cranial neural crest–like/odontogenic mesenchymal cells offer a renewable, standardized source that, when combined with the above maintenance conditions and epithelial partners, may further extend the durability and reproducibility of odontogenic capacity.

In conclusion, this study presents a novel approach to maintain the odontogenic potential of mDMCs in vitro and provides a comprehensive molecular characterization of the cells under different culture conditions. These findings lay the groundwork for future advances in tooth regeneration and offer new perspectives on the regulation of odontogenic potential in DMCs.

## Materials and methods

For detailed materials and methods, please refer to the supplementary material.

### Study design

This study was designed to investigate the long-term maintenance of the odontogenic potential of mDMCs in vitro. Unless otherwise noted, all in vitro experiments were repeated at least three times to ensure robust outcomes. Animals were randomly assigned to experimental groups in all cases. The experiments were not conducted in a blinded manner.

### Animal treatments

All animal experiments were performed in accordance with the updated ARRIVE 2.0 guidelines and approved by the Ethical Committee on Animal Experiments at Guangzhou Institutes of Biomedicine and Health, Chinese Academy of Sciences. The laboratory animal welfare and ethics code are N2022074 and IACUC2019041.

### Reconstitution of bioengineered tooth germs and subrenal capsule assays

For regenerative tooth germ experiments, a fragment of molar epithelium at E14.5-E16.5 embryos or a pellet of mRTECs was placed onto a mesenchymal specimen. For regenerative cell specimen preparation, 1.0 × 10^5^ cells were placed into a 1.5 mL EP tube, centrifuged at 6,600 rpm for 4.5 min, and then incubated at 37℃ for 1–2 h to form cell pellets. All bioengineered recombinants were cultured in a traditional organ culture system (Trowell-type system) with Dulbecco’s Modified Eagle Medium with High Glucose (DMEM-HG) supplemented with 10% FBS, 100 U/ml penicillin, and 100 μg/ml streptomycin for 1–2 days. They were then transplanted beneath the renal capsule of adult male ICR mice. Approximately eight recombinants were transplanted per kidney. Host mice were sacrificed after three weeks, and the kidneys with calcified tissues were harvested.

### Statistical analysis

The RNA-seq data processing and analysis were performed with Python version 3.8 (Python Software Foundation) and R version 3.5 (R Foundation). Normalization and differential expression analysis of the bulk RNA-seq data were performed using the DESeq2 R package (version 1.36.0). A general linear model (GLM) based on the negative binomial distribution was fitted to the bulk RNA-seq data, and statistical significance was tested using the likelihood ratio test (LRT). Benjamini–Hochberg multiple testing corrected *p* values with significance cut-off of 0.05 are reported as false discovery rate (FDR). When analyzing scRNA-seq data, we identified differentially expressed genes using the Wilcoxon rank sum test in Scanpy (version 1.9.5). Statistical analysis of qPCR data was performed using GraphPad Prism 9. The relative gene expression was calculated using the 2^(-ΔΔCt) method. For comparisons between two groups, an unpaired t-test was used. For comparisons of multiple treatment groups to a single control, a one-way ANOVA with Dunnett’s post-hoc test was performed. A two-way ANOVA with Bonferroni’s post-hoc test was performed to compare the two culture media across different time points. The minimum threshold for significance was defined as *p* < 0.05.

## Supplementary Information

Supplementary Material 1. Supplementary methods and figures.

## Data Availability

scRNA-seq and bulk RNA-seq data have been deposited in the SRA database as BioProject PRJNA851553.

## Abbreviations

DMCs: Dental mesenchymal cells
mDMCs: Mouse dental mesenchymal cells
mRTEC: Mouse renal tubular epithelial cell
FBS: Fetal bovine serum
KSR: Knockout serum replacement
Dox: Doxorubicin
DM: Dorsomorphin
RA: Retinoic acid
ATRA: All-trans retinoic acid
bulk RNA-seq: Bulk RNA sequencing
scRNA-seq: Single-cell RNA sequencing
DPSCs: Dental pulp stem cells
SHED: Stem cells from human exfoliated deciduous teeth
DFSCs: Dental follicle stem cells
PDLSCs: Periodontal ligament stem cells
HE: Hematoxylin–eosin staining
SA-β-gal: Senescence-associated beta-galactosidase
DEG: Differentially expressed gene
dp: Dental pulp
pD: Predentin
D: Dentin
es: Enamel space
Am: Ameloblast layer/ameloblasts
GO: Gene ontology
