# Supplementary material for: Regenerative teeth induced by in vitro mesenchymal cells in mice via repressing BMP4 and activating retinoic acid/osteopontin
Source: Cell Regen. 2025 Dec 5;14:51. doi: 10.1186/s13619-025-00271-9 (PMC12678684; doi:10.1186/s13619-025-00271-9)
Supplement: Supplementary file 1 — Supplementary Material 1. Supplementary methods and figures. [file 13619_2025_271_MOESM1_ESM.docx]

**Regenerative Teeth Induced by in vitro Mesenchymal Cells in Mice via Repressing BMP4 and activating Retinoic Acid/Osteopontin**

Shubin Chen, Yifan Zhao, Hongxing Chu, Qinxing Mo, Jiashu Zhang, Xiaoming Chen, Yanmei Zhang, Xiaomei Li, Di Wu, Pengfei Liu, Bo Feng, Dajiang Qin, Yaofeng Wang, Duanqing Pei, Jinglei Cai

**Supplementary Methods**

**Animal** **treatments**

Mouse embryos were from 8 to 10-week-old female ICR mice. All surgical procedures were performed under general anesthesia induced by intraperitoneal injection of 25 mL/kg of avertin (100 mL avertin containing 1.25 g 2,2,2-tribromoethanol, Sigma-Aldrich, Cat. No. T48402 and 2.5 mL 2-methyl-2-butanol, Sigma-Aldrich, Cat. No. 240486). Tooth germs from E14.5-E16.5 embryos were digested with 0.75 mg/mL Dispase II at 37℃ for 35-45 minutes. Dental mesenchyme and dental epithelium were separated using tungsten needles.

**Cell culture**

Mouse dental mesenchymal cells (mDMCs) were dissociated from dental mesenchyme at E14.5 and seeded on gelatin-coated cell plates. Cells were cultured in FBS-based medium or N2B27 medium. FBS medium consisted of high-glucose Dulbecco's Modified Eagle Medium (DMEM-HG, HyClone, Cat. No. SH30022.01) supplemented with 10% fetal bovine serum (FBS, Gibco, Cat. No. 10099141C), 100 U/ml penicillin, and 100 g/ml streptomycin. N2B27 medium contained 50% Dulbecco's modified eagle medium **:** nutrient mixture F-12 (DMEM/F-12, Gibco, Cat. No. C11330500BT) and 50% neurobasal™ medium (Gibco, Cat. No. 21103049), and supplemented with 200x N2 supplement (Gibco, Cat. No. 17502-048), 100x B27 supplement (Gibco, Cat. No. 17504-044), 20 ng/mL basic fibroblast growth factor (bFGF, PeproTech Cat. No. 450-33), and 20 ng/mL epidermal growth factor (EGF, PeproTech Cat. No. 315-09). FBS or N2B27 medium was changed every two and three days, respectively. Cells in both media were passaged upon reaching 90-100% confluence, which consistently occurred on day 7 (D7) after plating. Cells after the first replating were designated Passage 1 (P1). Mouse renal proximal tubular epithelial cells (mRTECs, ScienCell, Cat. No. M4100-57) were cultured in Roswell Park Memorial Institute (RPMI) 1640 Medium (Gibco, Cat. No. C11875500BT) supplemented with 10% FBS (Gibco, Cat. No. 10099141), 100 U/ml penicillin, and 100 µg/ml streptomycin (Gibco, Cat. No. 10378016) without coating.

**Cell viability assay**

mDMCs were seeded in 96-well plates at a density of 2.0 × 10^4^ cells/mL in a final volume of 0.1 mL per well. The cell proliferation rates at different culture times were subsequently assessed using the cell counting kit-8 (CCK-8, Beyotime, Cat. NO. C0037) according to the manufacturer's instructions. In brief, the cells in 96-well plate were treated with 20 μL CCK-8 solution, and incubated for 2 hours. The absorbance of each well was then quantified at 450 nm.

**Cell cycle analysis**

For cell cycle phase analysis, mDMCs were harvested and fixed in 70% cold ethanol overnight at −20°C. Fixed cells were subsequently washed in phosphate-buffered saline (PBS, Kinlogix, Cat. No. M1013). For staining of nuclei, cells were incubated with propidium iodide (50 μg/mL) (Sigma-Aldrich, Cat. No. P4864) in the dark for 30 minutes at 4°C. Stained cells were subsequently analyzed using a BD Accuri™ C6 Plus (BD Biosciences) flow cytometer. For each analysis, 10,000 events were evaluated with the software FlowJo (version 10.0.7).

**Senescence-associated β-galactosidase (SA-β-gal) staining**

mDMCs were plated 1.0 × 10^4^ cells/cm^2^ and assessed for SA-β-Gal activity at various times after incubation in FBS or N2B27 medium. The positive control was represented by the mDMCs incubated for 48 hours in complete medium with 500 nM doxorubicin (Dox, MedChem Express, Cat. No. HY-15142), and then cultured in complete medium for five days, while mDMCs treated with DMSO was used a negative control. SA-β-gal staining of the mDMCs were performed using a commercial SA-β-gal staining kit (Beyotime, Cat. No. C0602) according to the manufacture’s protocol. In brief, cells were fixed in the fixative solution for 15 minutes. After washing with PBS for three times, cells were incubated with SA-β-gal working solution at 37 °C for 16 hours. Ice-cold PBS was used to stop the enzymatic reaction.

**BMP signal of suppression and activation**

To examine BMP signaling on maintenance of the odontogenic potential in two medium systems, mDMCs were seeded at a density of 1.0 × 10^4^ cells/cm^2^. Dorsomorphin (DM, Sigma-Aldrich, Cat. No. P5499) was reconstituted in dimethyl sulfoxide (DMSO, Sigma-Aldrich, Cat. No. D2650), BMP4 protein (R&D System, Cat. No. 5020-BP) was reconstituted at sterile 4 mM HCl containing 0.1% bovine serum albumin and stored at −20 °C. mDMCs were cultured for four days in FBS medium supplemented with 2 μM DM (FBS+DM group) and an equivalent volume of DMSO as vehicle control(FBS group). mDMCs were cultured for four days in N2B27 medium supplemented with 25 ng/mL BMP4 protein (N2B27+BMP4 group) and an equivalent volume of BMP4 solvent as control (N2B27 group). The treated mDMCs were harvested for regenerative tooth analysis.

**OPN protein or ATRA treatment**

To examine the effect of osteopontin (OPN, MedChem Express, Cat. No. HY-P78358) or all trans retinoic acid (ATRA, Sigma-Aldrich, Cat. No. R2625) on maintenance of the odontogenic potential in FBS medium, mDMCs were seeded at a density of 1.0 × 10^4^ cells/cm^2^ and cultured in FBS medium. OPN protein was reconstituted in PBS, ATRA was reconstituted in DMSO; and both were stored at −20 °C. Working concentration of 1 μg/mL OPN protein (FBS + OPN group) or 10 μM ATRA (FBS + ATRA group) were added to FBS medium. Equivalent volume of PBS as control (FBS + PBS group) or DMSO (FBS + DMSO group) were added to FBS medium and used as a control respectively. After four days of treatment, mDMCs were then harvested for regenerative tooth analysis. The working concentrations of OPN protein (1 μg/mL) (Carvalho et al. 2020; Yuan et al. 2020; Lin et al. 2020) and ATRA (10 μM) (Zhang et al. 2025; Silvis et al. 2016; Horiguchi et al. 2014) were based on previous reports.

**Real-time quantitative PCR**

Real-time Polymerase Chain Reaction (qPCR) was performed as previously described (Zhao et al. 2024). Total RNA was extracted with Trizol (Invitrogen, Cat. No. 15596018CN). Then, 2 μg of RNA was reverse transcribed using a reverse transcription kit (Takara) and qPCR was performed using ChamQ SYBR qPCR Master Mix (Vazyme, Cat. No. Q311-02). Results were normalized to β-actin, and all assays were conducted in triplicate. Primer sequences (5’-3’) are listed below.

|  | Forward | Reverse |
| --- | --- | --- |
| *β-actin* | GGCTGTATTCCCCTCCATCG | CCAGTTGGTAACAATGCCATGT |
| *Lhx8* | ACACGAGCTGCTACATTAAGGA | CCAGTCAGTCGAGTGGATGTG |
| *Msx1* | TCATGGCCGATCACAGGAAG | GGAGTCCTCCGACTGAGAAATG |
| *Pax9* | CATTCGGCTTCGCATCGTG | CTCCCGGCAAAATCGAACC |
| *Bmp4* | TGTGAGGAGTTTCCATCACGA | CAGGAACCATTTCTGCTGGGG |
| *Barx1* | CGGAGTCGCACCGTATTCAC | TCTTCACCTGTAACTGGCTCA |
| *Dlx2* | CTACGGCACCAGTTCGTCTC | CCGTTCACTATTCGGATTTCAGG |
| *Spp1* | AGCAAGAAACTCTTCCAAGCAA | GTGAGATTCGTCAGATTCATCCG |
| *Cdkn1a* | CCTGGTGATGTCCGACCTG | CCATGAGCGCATCGCAATC |
| *Cdkn2a* | CGCAGGTTCTTGGTCACTGT | TGTTCACGAAAGCCAGAGCG |
| *Lmnb1* | CCGGCCTCAAGGCTCTCTA | TGCCGCCTCATACTCTCGAA |

**Semi-quantitative PCR**

Assays were performed in triplicate. Each reaction contained 2 µL of cDNA sample, 1 µL of each primer, 12.5 µL 2× Taq Master Mix (Vazyme, Cat. No. P111-02), and 8.5 µL ddH_2_O, in a final volume of 25 µL. The PCR conditions were: initial denaturation at 95 °C for three minutes, followed by 35 cycles of 95 °C for 15 seconds, and a combined annealing and extension step at 61 °C for 35 seconds. The PCR products were electrophoresed on 2.0% agarose gels, stained with StarGreen safe Nucleic Acid Dye (GenStar, Cat. No. ZE111-101), and photographed. Primer sequences (5’-3’) are listed below.

|  | Forward | Reverse |
| --- | --- | --- |
| *Gapdh* | AGTGTTTCCTCGTCCCGTAG | GCCCTTCCACAATGCCAAAG |
| *Npsh1* | ATGGGAGCTAAGGAAGCCACA | GATGGAGAGGATTACGCTGGG |
| *Pitx2* | ACCCCGGCTATTCGTACAAC | GAGGACAGGGGATTGACGTTC |
| *Fgf8* | CCGAGGAGGGATCTAAGGAAC | CTTCCAAAAGTATCGGTCTCCAC |
| *Msx1* | TGCTGCTATGACTTCTTTGCC | GCTTCCTGTGATCGGCCAT |
| *Krt8* | AAGTTCGTGCCCAGTACGAG | GCTTCCCATCTCGGGTTTCA |

**Bulk RNA sequencing (Bulk RNA-seq) and data analysis**

Cells were collected and lysed with 1 mL Trizol (Invitrogen). Total RNA was extracted by the [TRIzol](https://www.sciencedirect.com/topics/biochemistry-genetics-and-molecular-biology/trizol) method, and RNA quality was evaluated using the Bioanalyzer 2100 (Agilent, Santa Clara). Sequencing libraries were constructed followed by the standard Illumina protocols, and sequenced on the Illumina NovaSeq 6000 platform with paired-end reads of 150 bp in Annoroad Gene Technology. Each group was performed three biological replicates. The filtered sequencing reads were mapped to the Mus musculus GRCm38.102 genome using Hisat2 (v2.1.0) (Siren et al. 2014) software with the default parameter. The [gene expression level](https://www.sciencedirect.com/topics/biochemistry-genetics-and-molecular-biology/gene-expression-level) was normalized using the transcripts per million (TPM) (Li and Dewey 2011). DESeq2 (v1.36.0) package (Love et al. 2014) was used to identified differentially expressed genes (DEGs).  In differential expression profiles analysis, up-regulated genes were defined as those with over 2-fold alterations, and the down-regulated genes were defined as those with less than half-fold alterations.

**Single-cell RNA sequencing (scRNA-seq)**

Cell concentration was determined using a hemocytometer, and adjusted to obtain the target concentration for the 10x [Chromium](https://www.sciencedirect.com/topics/biochemistry-genetics-and-molecular-biology/chromium) chip loading. The cells were loaded into the 10x Chromium Controller, and the Single Cell 3′ Reagent Kit v2 was used according to the manufacturer’s protocol. Following library preparation and quantitation, the libraries were sequenced on the Illumina HiSeq X Ten platform. The sequencing of libraries was performed by Annoroad Gene Technology Co., Ltd, Beijing, China.

**scRNA-seq data analysis**

scRNA-seq data were pre-processed using a 10x Genomics Cell Ranger pipeline. Briefly, reads were aligned to the mouse genome (mm 10) with the setting ‘--r1-length=26 --r2-length=98’. Default cellranger parameters for 10x Genomics were used to obtain the UMI count matrix. Downstream analysis was performed with Scanpy (Wolf et al. 2018). The UMI count matrix was lightly filtered to exclude cell barcodes with low numbers of counts. Cells with less than 200 genes detected or more than 5% fraction of mitochondrial or beta-globin counts were removed. Genes with less than three cells detected were removed. Cell cycle genes were regressed out. The Gene Ontology (GO) analysis was performed using clusterProfiler (Yu et al. 2012). Gene regulatory network (GRN) analysis was performed using pySCENIC (Van de Sande et al. 2020). Data visualizations were performed using Seurat (Stuart et al. 2019) or Scanpy (Wolf et al. 2018). RNA velocity analysis (La Manno et al. 2018) was performed using scVelo (Bergen et al. 2020) and dynamo (Qiu et al. 2022) packages in python.

**Histological analysis for tissue sections**

Mouse samples were fixed in 4% paraformaldehyde (PFA) for 24 hours, and decalcified with 10% EDTA for three weeks. Paraffin sections with 7-μm thickness were stained with standard hematoxylin and eosin (HE) (C0105, Beyotime) according to the manufacturer’s instructions.

**Supplementary figure legends**


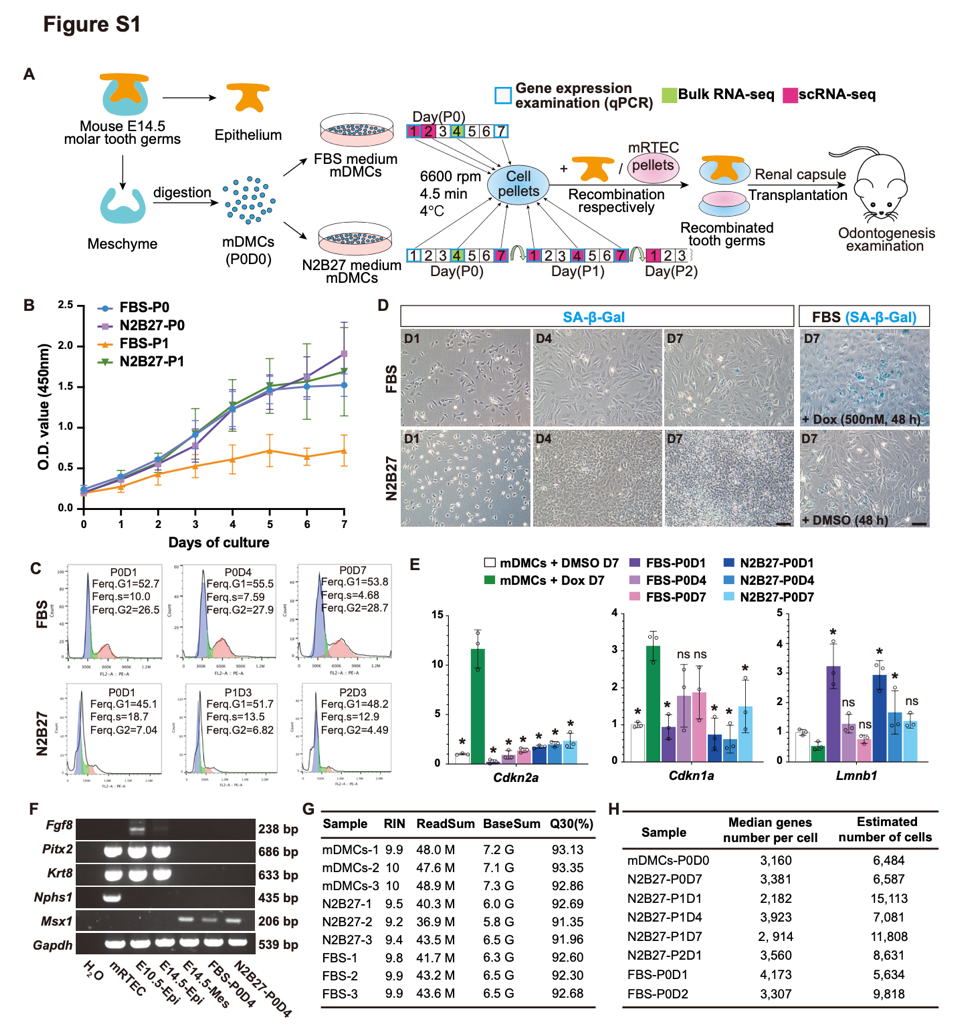


**Figure S1**

(A) Schematic of experimental workflow. mDMCs isolated from molar tooth germs at E14.5 were cultured and further examined for the odontogenic potential using two culture systems to identify the *in vitro* maintenance of their odontogenic capacity. With qPCR, bulk RNA-seq, scRNA-seq, tissue recombination and renal capsule transplantation, molecular mechanisms underlying maintenance of the odontogenic potential in mDMCs were investigated.

(B) The viability and proliferation of mDMCs in FBS medium or N2B27 medium before (P0) and after passage (P1) as determined by CCK-8 assay.

(C) Representative flow cytometry histograms of cell cycle distribution after PI staining. mDMCs cultured in FBS medium for one day (P0D1), four days (P0D4) and seven days (P0D7) and mDMCs cultured in N2B27 medium for one day (P0D1), three days after first passage (P1D3), and three days after second passage (P2D3) were identified. The peaks corresponding to the G0/G1, S, and G2/M phases are indicated. The experiment was only done for one time.

(D-E) Identification of senescence-associated secretory phenotype (SASP) in the cutured mDMCs in FBS medium or N2B27 medium by SA-β-galactosidase staining and qPCR with positive markers (*Cdkn1a* and *Cdkn2a*) and negative marker (*Lmnb1*). Positive control group, mDMCs treated with Dox; negative control group, mDMCs treated with DMSO.

(F) Semi-quantitative PCR results of the odontogenic or nonodontogenic genes in the specimens from mouse renal proximal tubular epithelial cells (mRTECs), presumptive dental epithelia at E10.5 (E10.5-Epi), dental epithelia at E14.5 (E14.5-Epi), dental mesenchyme at E14.5 (E14.5-Mes), mDMCs cultured for four days in FBS medium (FBS-P0D4) or N2B27 medium (N2B27-P0D4).

(G) Samples information of bulk RNA-seq. The samples were divided into three groups of three biological replicates each. Mouse dental mesenchymal cells (mDMCs) from molar tooth germs at E14.5 as a control group. mDMCs were cultured for four days in N2B27 medium as the N2B27 group (N2B27) and in FBS medium for four days as the FBS group (FBS). RNA integrity number (RIN), total raw sequencing reads (ReadSum), total raw sequencing bases (BasesSum). The percentage of bases with a quality score greater than or equal to 30 [Q30(%)].

(H) Detected cells and genes from scRNA-seq. Sample name: mDMCs  from the lower first molar tooth germs at E14.5 as a control group (mDMCs-P0D0), mDMCs were cultured for seven days at P0 (without passage) (N2B27-P0D7), one day at P1 (after first passage) (N2B27-P1D1), four days at P1 (N2B27-P1D4), seven days at P1 (N2B27-P1D7) , one day at P2 (after second passage) (N2B27-P2D1) in N2B27 medium and in FBS medium for one day at P0 (FBS-P0D1), and two days at P0 (FBS-P0D2).

**References for supplementary methods**

Carvalho MS, Silva JC, Hoff CM, Cabral JMS, Linhardt RJ, da Silva CL, et al. Loss and rescue of osteocalcin and osteopontin modulate osteogenic and angiogenic features of mesenchymal stem/stromal cells. J Cell Physiol. 2020;235(10):7496-7515. doi:10.1002/jcp.29653

Yuan Y, Liu Q, Wu Z, Luo W. Mechanistic Insight on the Interaction between OPN and Integrin alphanubeta3 in Osteoarthritis. Biomed Res Int. 2020;2020:2905634. doi:10.1155/2020/2905634

Lin R, Wu S, Zhu D, Qin M, Liu X. Osteopontin induces atrial fibrosis by activating Akt/GSK-3beta/beta-catenin pathway and suppressing autophagy. Life Sci. 2020;245:117328. doi:10.1016/j.lfs.2020.117328

Zhang R, Tang T, Zhuang H, Wang P, Yu H, Xu H, et al. Temperature-sensitive sodium beta-glycerophosphate/chitosan hydrogel loaded with all-trans retinoic acid regulates Pin1 to inhibit the formation of spinal cord injury-induced rat glial scar. Bioeng Transl Med. 2025;10(3):e10729. doi:10.1002/btm2.10729

Silvis AM, McCormick ML, Spitz DR, Kiningham KK. Redox balance influences differentiation status of neuroblastoma in the presence of all-trans retinoic acid. Redox Biol. 2016;7:88-96. doi:10.1016/j.redox.2015.11.012

Horiguchi M, Kojima H, Sakai H, Kubo H, Yamashita C. Pulmonary administration of integrin-nanoparticles regenerates collapsed alveoli. J Control Release. 2014;187:167-74. doi:10.1016/j.jconrel.2014.05.050

Zhao Y, Chen S, Liu X, Chen X, Yang D, Zhang J, et al. Single-cell RNA-seq of in vitro expanded cells from cranial neural crest reveals a rare odontogenic sub-population. Cell Prolif. 2024;57(6):e13598. doi:10.1111/cpr.13598

Siren J, Valimaki N, Makinen V. Indexing Graphs for Path Queries with Applications in Genome Research. IEEE/ACM Trans Comput Biol Bioinform. 2014;11(2):375-88. doi:10.1109/TCBB.2013.2297101

Li B, Dewey CN. RSEM: accurate transcript quantification from RNA-Seq data with or without a reference genome. BMC Bioinformatics. 2011;12:323. doi:10.1186/1471-2105-12-323

Love MI, Huber W, Anders S. Moderated estimation of fold change and dispersion for RNA-seq data with DESeq2. Genome Biol. 2014;15(12):550. doi:10.1186/s13059-014-0550-8

Wolf FA, Angerer P, Theis FJ. SCANPY: large-scale single-cell gene expression data analysis. Genome Biol. 2018;19(1):15. doi:10.1186/s13059-017-1382-0

Yu G, Wang L-G, Han Y, He Q-Y. clusterProfiler: an R package for comparing biological themes among gene clusters. Omics : a journal of integrative biology. 2012;16(5):284-287. doi:10.1089/omi.2011.0118

Van de Sande B, Flerin C, Davie K, De Waegeneer M, Hulselmans G, Aibar S, et al. A scalable SCENIC workflow for single-cell gene regulatory network analysis. Nat Protoc. 2020;15(7):2247-2276. doi:10.1038/s41596-020-0336-2

Stuart T, Butler A, Hoffman P, Hafemeister C, Papalexi E, Mauck WM, III, et al. Comprehensive integration of single-cell data. Cell. 2019;177(7):1888-1902.e21. doi:10.1016/j.cell.2019.05.031

La Manno G, Soldatov R, Zeisel A, Braun E, Hochgerner H, Petukhov V, et al. RNA velocity of single cells. Nature. 2018;560(7719):494-498. doi:10.1038/s41586-018-0414-6

Bergen V, Lange M, Peidli S, Wolf FA, Theis FJ. Generalizing RNA velocity to transient cell states through dynamical modeling. Nat Biotechnol. 2020;38(12):1408-1414. doi:10.1038/s41587-020-0591-3

Qiu X, Zhang Y, Martin-Rufino JD, Weng C, Hosseinzadeh S, Yang D, et al. Mapping transcriptomic vector fields of single cells. Cell. 2022;185(4):690-711.e45. doi:<https://doi.org/10.1016/j.cell.2021.12.045>
